# High-order femtosecond vortices up to the 30th order generated from a powerful mode-locked Hermite-Gaussian laser

**DOI:** 10.1038/s41377-023-01241-z

**Published:** 2023-08-30

**Authors:** Hongyu Liu, Lisong Yan, Hongshan Chen, Xin Liu, Heyan Liu, Soo Hoon Chew, Alexander Gliserin, Qing Wang, Jinwei Zhang

**Affiliations:** 1 School of Optical and Electronic Information and Wuhan National Laboratory for Optoelectronics, Huazhong University of Science and Technology, Wuhan, 430074 China; 2 Department of Optics and Mechatronics Engineering, College of Nanoscience and Nanotechnology, Pusan National University, Busan, 46241 South Korea; 3 Max Planck Center for Attosecond Science, Max Planck POSTECH/Korea Research Initiative, Pohang, 37673 South Korea; 4 School of Optics and Photonics, Beijing Institute of Technology, Beijing, 100081 China

**Keywords:** Ultrafast lasers, Mode-locked lasers, Solid-state lasers

## Abstract

Femtosecond vortex beams are of great scientific and practical interest because of their unique phase properties in both the longitudinal and transverse modes, enabling multi-dimensional quantum control of light fields. Until now, generating femtosecond vortex beams for applications that simultaneously require ultrashort pulse duration, high power, high vortex order, and a low cost and compact laser source has been very challenging due to the limitations of available generation methods. Here, we present a compact apparatus that generates powerful high-order femtosecond vortex pulses via astigmatic mode conversion from a mode-locked Hermite-Gaussian Yb:KGW laser oscillator in a hybrid scheme using both the translation-based off-axis pumping and the angle-based non-collinear pumping techniques. This hybrid scheme enables the generation of femtosecond vortices with a continuously tunable vortex order from the 1st up to the 30th order, which is the highest order obtained from any femtosecond vortex laser source based on a mode-locked oscillator. The average powers and pulse durations of all resulting vortex pulses are several hundred milliwatts and <650 fs, respectively. In particular, 424-fs 11th-order vortex pulses have been achieved with an average power of 1.6 W, several times more powerful than state-of-the-art oscillator-based femtosecond vortex sources.

## Introduction

Over the past three decades, the development of optical vortices (OVs) has been of considerable research interest driven by their distinctive properties, such as helical phase front and orbital angular momentum. Substantial progress has been made with continuous-wave and long-pulse optical vortices in aspects ranging from fundamental theories to the generation mechanism and technologies for improving tunability^1^ with various impactful applications in optical communication^2,3^, quantum entanglement^4,5^, and optical tweezers^6^. Compared to the continuous-wave and long-pulse optical vortices, femtosecond optical vortices (FOVs) not only possess helical phase fronts in the transverse modes, but also exhibit a fixed phase difference between the longitudinal modes. This multi-dimensional control of femtosecond laser pulses with high peak power is crucial for numerous applications such as launching and steering femtosecond micro/nano manipulation^7^, exploring the dynamics of the interaction between matter and complex light fields^8^, and pump-probe spectroscopy^9^, as well as for nonlinear vortex optics^10,11^ and strong-field physics^12^. All these advanced applications will greatly benefit from a FOV laser source that combines a high and tunable vortex order, high average power and femtosecond pulse duration.

Traditional methods for generating optical vortices rely on the spatial phase modulation of Gaussian beams via phase-modulation components such as spiral phase plates^13^, metasurfaces^14,15^, computer-generated holograms^16^ and spatial light modulators^17^. These methods are effective for nanosecond or picosecond pulsed vortices, but will introduce strong spatial dispersion in the case of femtosecond vortices with a broad spectral bandwidth^18^. Additional dispersion compensation elements are therefore required at the expense of complexity to maintain the phase relationship of different wavelengths with their phase singularities in order to minimize the pulse duration^19^. In addition, the use of phase-modulation components typically suffers from a low conversion efficiency and a low damage threshold of the elements, which severely limits the power scaling of the femtosecond vortex pulses. Another method to generate FOV is to use optical parametric amplification or optical parametric chirped pulse amplification, with the vortex characteristic induced by phase components prior to amplification. This method has enabled FOVs with very short pulse durations (from ~10 fs to ~100 fs) and pulse energies up to mJ level^20–24^. However, the beam quality and stability cannot be ensured, and high-order FOVs are hard to be obtained.

Recent alternatives to these approaches demonstrate direct generation of femtosecond vortices with high beam quality from solid-state mode-locked laser oscillators by employing defect-spot mirrors^25^, off-axis pumping with optimized cavity alignment^26^. However, these femtosecond vortex lasers can only deliver Laguerre-Gaussian (LG) modes with a topological charge of one, and produce average powers of few tens of milliwatts at best. In contrast to LG modes, Hermite-Gaussian (HG) modes are more likely to be excited in a laser cavity since the cavity usually exhibits rectangular symmetry when the surfaces of the optical elements are tilted with respect to the laser beams^27^. A solid-state mode-locked HG laser oscillator inherently allows for the generation of high-power, femtosecond, high-order transverse HG modes, which can be conveniently and efficiently converted into the corresponding LG modes by using a simple astigmatic mode converter (AMC)^28^. In order to excite the femtosecond HG modes inside a laser cavity, two approaches have been investigated which utilize translation-based off-axis pumping and angle-based non-collinear pumping techniques^29,30^, respectively. For instance, with translation-based off-axis pumping, 20-ps vortex pulses with a topological charge up to the 9th order were achieved from a self-mode-locked Nd:GdVO_4_ oscillator^31^, and 109-fs LG_01_ vortex pulses were obtained from a 2-μm laser oscillator mode-locked with a semiconductor saturable absorption mirror (SESAM)^32^. The second approach has led to the generation of high-power femtosecond LG_01_ and LG_02_ vortex pulses at 2 μm from a SESAM mode-locked Tm:CYA oscillator^30^. In addition, femtosecond vortex pulses from LG_01_ to LG_04_ were realized using a SESAM mode-locked Yb:CALGO oscillator^33^. These results have shown a great potential of the mode-locked HG oscillator scheme for generating superior FOVs; however, it is still very challenging to further increase the order tunability and realize higher average power in the femtosecond regime.

**Fig. 1:**
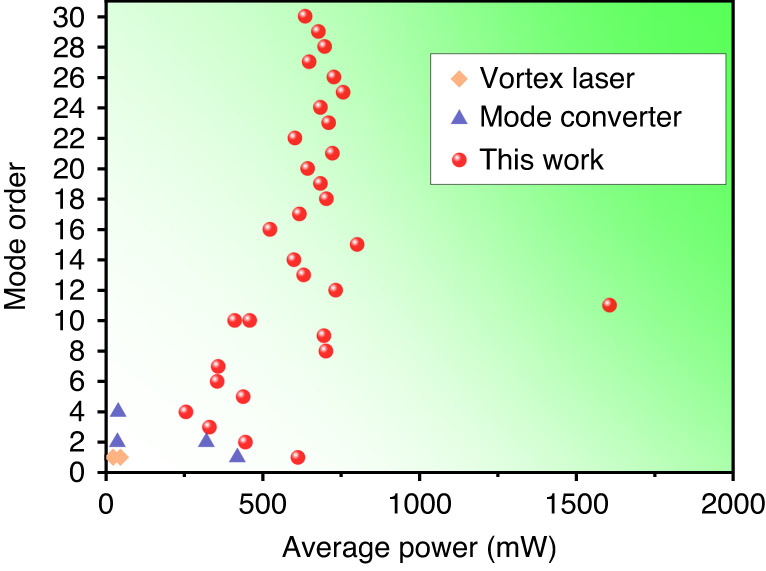
Overview of state-of-the-art femtosecond vortices based on solid-state mode-locked laser oscillators^25,26,30,33,41^. The powers reported for mode converters (blue triangles) refer to HG modes before the conversion; the powers reported for vortex lasers (yellow diamonds) and this work (red circles) refer to LG modes after the conversion

In this work, we present for the first time a mode-locked HG Yb:KGW laser oscillator in a hybrid scheme combining both the translation-based off-axis pumping and the angle-based non-collinear pumping techniques. The LG vortex pulses were obtained by converting the HG modes in an AMC set-up. Our approach yields the widest range of order tuning—from the 1st up to the 30th order—for femtosecond vortices generated from HG mode-locked oscillators (Fig. 1). The pulses at all vortex orders have average powers of several hundred milliwatts and pulse durations <650 fs. By optimizing the parameters of the oscillator, we could even generate 1.6-W FOV pulses at the 11th order, which is the highest average power of any femtosecond vortex source based on mode-locked oscillators.

## Results

### Experimental setup

**Fig. 2:**
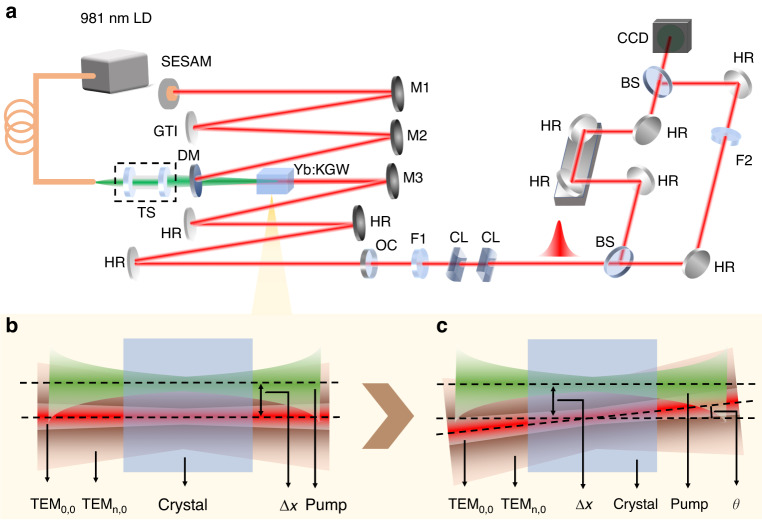
Schematic of the experiment. **a** Experimental setup. LD: laser diode; TS: telescope system; DM: dichroic mirror; HR: high-reflectivity mirror; M1, M2 and M3: concave mirrors with a radius of curvature of −150 mm, −200 mm and −200 mm, respectively; OC: output coupler with a transmission of 3%; F1, F2: lenses with a focal length of 150 mm and 88 mm, respectively; CL: cylindrical lenses; BS: beam splitter. **b** Schematic of translation-based off-axis pumping; **c** Schematic of hybrid angle-based non-collinear pumping

The schematic of the experimental setup is depicted in Fig. 2. The initial HG pulses were produced by a SESAM mode-locked Yb:KGW oscillator that operated at a repetition rate of 114 MHz. The pump beam was generated from a fiber-coupled laser diode at 981 nm, and focused into the laser crystal by a telescope system with a focused beam diameter of 105 μm. The fiber end and the telescope system were placed on a three-dimensional translation stage so that the pump beam can be shifted relative to the laser beam axis. A Z-shaped cavity was designed with a beam waist diameter of ~100 μm for the fundamental laser mode inside the crystal. The round-trip group delay dispersion (GDD) was −4000 fs^2^, provided by a GTI (Gires-Tournois interferometer) mirror.

### Generation of femtosecond HG modes from the 1st to the 10th order with translation-based off-axis pumping scheme

**Fig. 3:**
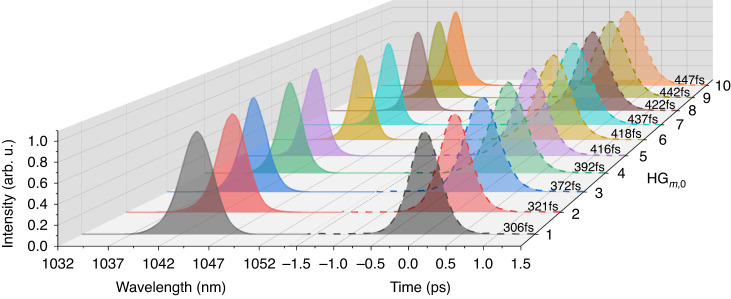
Spectra and autocorrelation traces for HG_1,0_ to HG_10,0_ femtosecond pulses with the translation-based off-axis pumping approach. The spectra were measured by an optical spectrum analyzer (DEVISER, AE8600) with a resolution of 0.5 nm

We first implemented the translation-based off-axis pumping scheme to generate tunable high-order HG modes. The oscillator initially operated under an ideal mode-overlapping condition and emitted a fundamental-mode laser beam. An off-axis displacement *∆x* was then introduced between the pump beam axis and the fundamental laser beam axis through shifting the translation stage gradually (Fig. 2b). By increasing *∆x*, HG_*m*,0_ modes with the order tunable from *m* = 1 to *m* = 10 were excited. Compared to the previous HG oscillators using a translation-based off-axis pumping scheme^32^, the oscillator we designed in this work had larger beam sizes for both the pump and laser beams. It enabled the generation of high-order HG modes with high power, which was essential for the realization of longitudinal mode locking. In addition, the larger beam size allowed the fine tuning of the overlapping condition between the pump and laser beam, which improved the spatial intensity distribution of the laser modes. By combining the SESAM technology (which is different from the self-mode locking technique in ref. ^31^), longitudinal mode-locking was automatically achieved for each order. This is, to our knowledge, the highest order ever achieved for femtosecond HG pulses from a passively mode-locked solid-state oscillator using the translation-based off-axis pumping scheme. The mode locking at each order was stable without any Q-switching behavior. Figure 3 shows the measured spectra and pulse durations, where a clear trend of pulse duration lengthening accompanied by spectral narrowing can be observed with increasing HG order. The average output powers were between 277 mW and 792 mW, and the optical-to-optical efficiency decreased roughly with increasing mode order (Table 1). The observed trends of the pulse duration and efficiency result from increased diffraction losses inside the cavity at higher mode orders.

**Table 1:** Summary of the parameters of femtosecond HG and LG pulses

Mode	*λ* (nm)	*P*_Pump_ (W)	*P*_HG_ (mW)	*P*_LG_ (mW)	*τ* (fs)	*η*_O-O_
HG_1,0_	1043.7	4.34	686	612	306	15.8%
HG_2,0_	1043.2	4.34	490	445	321	11.3%
HG_3,0_	1041.5	5.12	378	330	372	7.4%
HG_4,0_	1041.8	5.32	277	255	392	5.2%
HG_5,0_	1041.0	7.59	508	438	416	6.7%
HG_6,0_	1043.0	7.10	405	355	418	5.7%
HG_7,0_	1043.1	7.55	400	358	437	5.3%
HG_8,0_	1043.6	8.76	792	701	422	9.0%
HG_9,0_	1043.2	9.88	785	695	442	7.9%
HG_10,0_ (a)	1042.6	11.06	468	411	447	4.2%
HG_10,0_ (b)	1042.0	10.41	527	459	399	5.1%
HG_11,0_	1044.6	13.35	1824	1604	424	13.7%
HG_12,0_	1043.1	12.78	792	732	516	6.2%
HG_13,0_	1041.9	12.27	699	630	490	5.7%
HG_14,0_	1041.5	11.78	674	599	489	5.7%
HG_15,0_	1042.9	13.17	872	801	493	6.7%
HG_16,0_	1041.5	13.01	594	523	532	4.6%
HG_17,0_	1041.2	13.83	711	617	544	5.1%
HG_18,0_	1041.2	15.01	781	703	584	5.2%
HG_19,0_	1041.9	13.05	752	684	495	5.8%
HG_20,0_	1041.9	15.56	723	643	472	4.6%
HG_21,0_	1042.1	16.52	831	722	433	5.1%
HG_22,0_	1041.3	17.62	690	602	577	3.9%
HG_23,0_	1041.9	16.36	790	710	560	4.8%
HG_24,0_	1041.5	16.39	772	684	522	4.7%
HG_25,0_	1041.8	17.41	842	756	510	4.8%
HG_26,0_	1041.8	18.58	843	727	525	4.5%
HG_27,0_	1041.4	17.41	742	648	620	4.3%
HG_28,0_	1041.3	17.78	832	697	550	4.7%
HG_29,0_	1041.6	17.81	823	677	551	4.6%
HG_30,0_	1041.3	18.22	727	635	575	4.0%

HG_10,0_ (a) and HG_10,0_ (b) refer to the results from the translation-based off-axis pumping approach and the hybrid angle-based non-collinear pumping approach, respectively. *λ*: central wavelength; *P*_Pump_: pump power; *P*_HG_: average power of the generated HG modes from the oscillator; *P*_LG_: average power of the LG modes after the conversion from HG modes; *τ*, pulse width of the HG modes; *η*_O-O_: optical-to-optical efficiency from the pump laser to the generated HG laser. The power-stability measurement shows a typical deviation of <2% for all modes (Fig. S3(b))

**Fig. 4:**
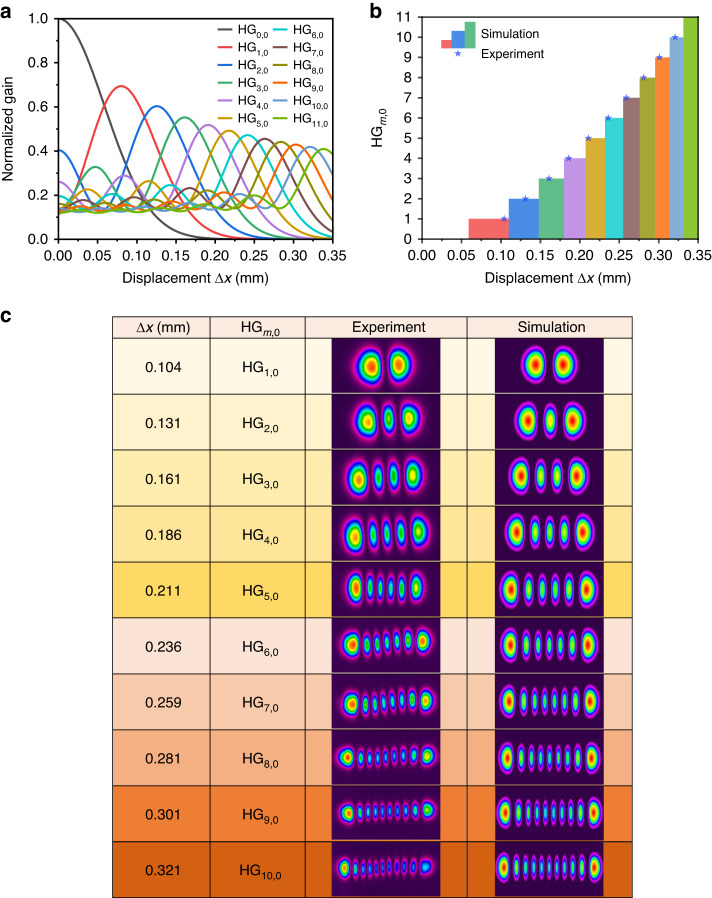
Simulation and experimental results for the HG_*m*,0_ modes from *m* = 1 to *m* = 10. **a** Simulation of the gain distribution of the HG_*m*,0_ modes from *m* = 0 to *m* = 11 as a function of the displacement $\varDelta{x}$, which is the offset between the pump beam axis and the fundamental-mode beam axis. **b** Comparison of the experimental $\varDelta{x}$ and mode orders with the simulated results. **c** Measured and simulated beam profiles for HG_1,0_ to HG_10,0_ femtosecond pulses with the translation-based off-axis pumping approach

A numerical simulation according to the model presented in ref. ^34^ was performed to elucidate the relationship between the HG_*m*,0_ modes and the displacement *∆x* under a constant pumping density. As shown in Fig. 4a, HG modes with successively higher-order dominate the gain distribution with increasing *∆x*. This can be explained by the fact that higher-order modes exhibit a larger beam size and thus a better overlap with the pump beam at larger *∆x*. When a mode of any order *m* has its highest gain, it suppresses the excitation of other modes, resulting in a pure HG_*m*,0_ mode generation with negligible contributions from other orders. The simulation also indicates a tendency of decreased total gain for higher-order HG modes when a certain HG_*m*,0_ mode dominates, which is attributed to the increased diffraction loss in the laser cavity. Figure 4b shows the displacement value of *∆x* for each HG mode order generated in the experiment, revealing an excellent agreement with the simulation results. The beam profile of the HG pulses was measured with a charge-coupled device beam profiler for each order at the corresponding *∆x*, matching well with the simulation (Fig. 4c).

### Generation of femtosecond HG modes from the 10th to the 30th order with the hybrid scheme

**Fig. 5:**
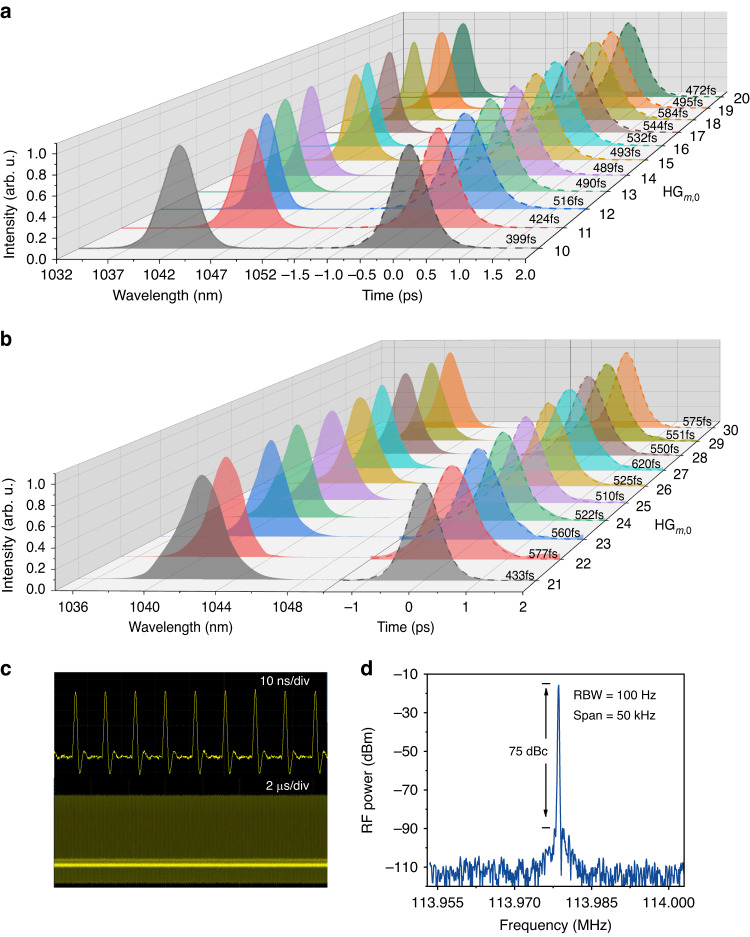
Characterization of the generated femtosecond HG modes. **a** Spectra and autocorrelation traces for HG_10,0_ to HG_20,0_ femtosecond pulses generated with the hybrid angle-based non-collinear pumping approach. **b** Spectra and autocorrelation traces for HG_21,0_ to HG_30,0_ femtosecond pulses. **c** Pulse trains of femtosecond HG_30,0_ pulses on different time scales. **d** RF spectrum of femtosecond HG_30,0_ pulses, showing a high signal-to-noise ratio of ~75 dB with a resolution bandwidth (RBW) of 100 Hz

A further increase of *∆x* reduced the total gain for HG_*m*,0_ modes with an order of *m* ≥ 11 to the point where the achievable intracavity power became insufficient for stable and tunable mode locking under the same cavity configuration. In order to generate femtosecond HG pulses of even higher orders, we introduced an angle of *θ* between the pump and the laser beam axes by adjusting the OC while still maintaining the off-axis displacement *∆x* (Fig. 2c). In comparison to the translation-based off-axis pumping and the traditional non-collinear pumping scheme, this hybrid approach relies on the interworking of both *∆x* and *θ*, which results in a precisely regulated and larger overlap of higher-order HG modes with the gain volume formed by the pump beam, thus it is beneficial for longitudinal mode locking. In order to start with a relatively high total gain, the displacement of the pump beam was reverted to optimally generate the HG_9,0_ mode (*∆x* = 0.301 mm). By changing *θ*, HG_*m*,0_ modes tunable from *m* = 10 to *m* = 20 can be generated, which are longitudinally mode locked by the SESAM. As in the case of translation-based off-axis pumping, the mode locking was self-started and stable without Q-switching. The spectra and autocorrelation traces of the pulses for each order are shown in Fig. 5a, revealing pulse durations below 600 fs in all cases. The achieved average powers of the femtosecond pulses were higher than 500 mW for all orders at higher pump powers (Table 1). Notably, the HG_10,0_ pulses exhibited a better performance in terms of average power, pulse duration and efficiency than pulses of the same order using the translation-based off-axis pumping scheme, owing to the relatively larger overlap with the pump volume and the resulting higher gain. Furthermore, a record-high average power of up to 1.824 W at a pump power of 13.35 W was achieved for the HG_11,0_ mode, resulting in a slightly shifted central wavelength around 1045 nm and a 424-fs pulse duration. The average power stability of the femtosecond HG_11,0_ mode was measured within one hour (Fig. S3a) and showed a root mean square (RMS) deviation of ~0.87%. For the HG_20,0_ mode, 723-mW pulses were realized with a pulse duration of 472 fs (sech^2^ fit).

In order to achieve longitudinal mode locking at higher-order transverse HG modes, we experimentally shifted the pump beam to a new position with *∆x* = 0.371 mm. The required non-collinear angle *θ* for the generation of femtosecond HG_21,0_ mode was then reduced correspondingly to balance the influence brought by the excess *∆x*. Femtosecond HG_*m*,0_ modes tunable from *m* = 21 to *m* = 30 were obtained by increasing the angle *θ* again with average powers higher than 500 mW in all cases (Table 1). Figure 5b shows the spectra and autocorrelation traces of the pulses for each order with pulse durations all below 650 fs. The highest practically achievable mode of HG_30,0_ yielded 727-mW pulses with a pulse duration of 575 fs (sech^2^ fit). The corresponding time bandwidth product is 0.34, which is close to the Fourier transform limit. The pulse train and the radio frequency (RF) spectrum for HG_30,0_ pulses are shown in Fig. 5c and d, respectively, indicating a stable mode locking. The calculated RMS value of the relative intensity noise within the range of 10 Hz and 2 MHz is about 0.43% relative to the electric power background (Fig. S4). The generation of higher-order femtosecond HG_*m*,0_ modes with *m* > 30 is currently limited by the available pump power which will be increased in the future.

**Fig. 6:**
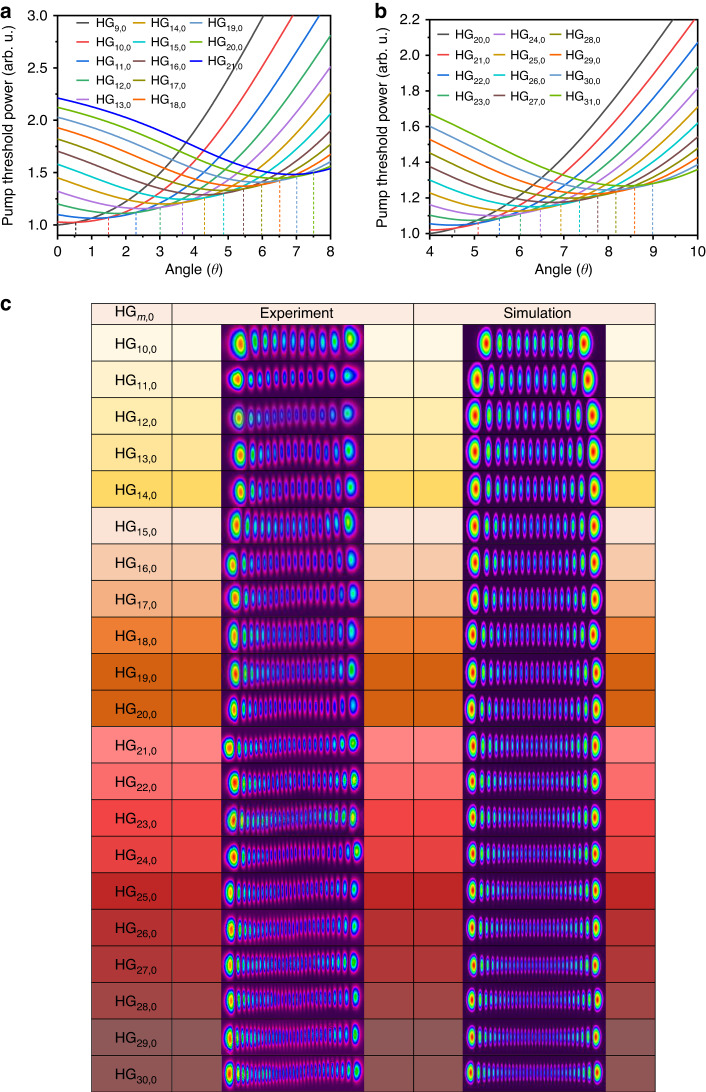
Simulation and experimental results for the HG_*m*,0_ modes from *m* = 10 to *m* = 30. **a** Simulation of the pump threshold for HG_*m*,0_ modes from *m* = 9 to *m* = 21 as a function of the angle *θ* between the pump beam axis and the fundamental-mode beam axis at a fixed off-axis displacement ($\varDelta{x}$ = 0.301 mm). **b** Simulation of the pump threshold for HG_*m*,0_ modes from *m* = 20 to *m* = 31 as a function of *θ* at a fixed off-axis displacement ($\varDelta{x}$ = 0.371 mm). **c** Measured and simulated beam profiles for HG_10,0_ to HG_30,0_ femtosecond pulses generated with the hybrid angle-based non-collinear pumping approach

A numerical model has been developed to analyze the pump threshold of the HG_*m*,0_ modes as a function of the angle *θ* in the case of a purely angle-based non-collinear scheme^30^. We optimized this model and introduced the off-axis displacement *∆x* as an additional variable into the calculation. Figure 6a, b shows the pump threshold of the different HG_*m*,0_ modes as a function of the angle with a fixed off-axis displacement of *∆x* = 0.301 mm (initial *θ* = 0°, starting from the HG_9,0_ mode) and *∆x* = 0.371 mm (initial *θ* = 4°, starting from the HG_20,0_ mode), respectively. With increasing *θ*, the HG mode of successively higher order exhibits the lowest pump threshold compared to the adjacent-order modes. This mode is the first to start stimulated emission and therefore dominates the lasing process. However, the lowest pump threshold increases with the mode order (and therefore with *θ*), indicating that a higher pump power is needed to excite higher-order modes. In the experiment, however, it was difficult to measure the exact value of the angle *θ*. The beam profiles of the HG modes from HG_10,0_ to HG_30,0_ measured after mode locking at the corresponding angle *θ* are shown in Fig. 6c, revealing an excellent agreement with the simulated profiles.

### Theoretical analysis and comparison of different pumping schemes

**Fig. 7:**
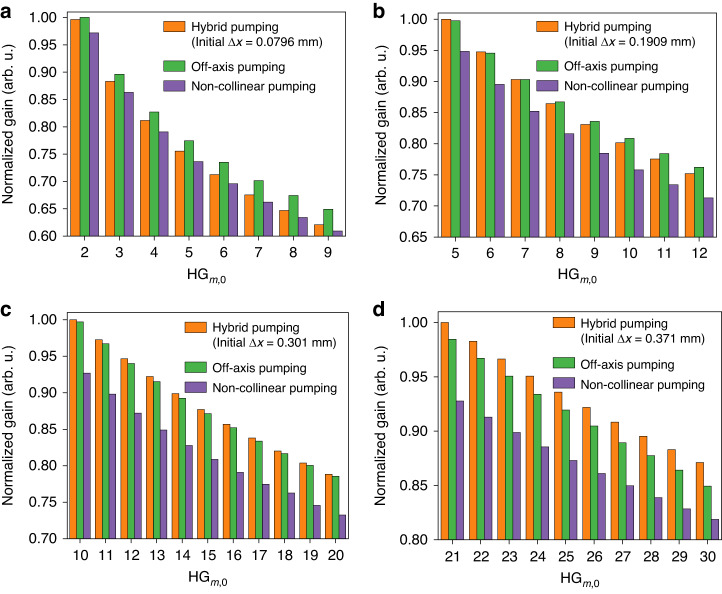
Simulation and comparison of the gain achieved between the pure translation-based off-axis pumping scheme, the pure angle-based non-collinear pumping scheme, and the hybrid pumping scheme for the HG_*m*,0_ modes. **a** For low order HG modes generation with $\varDelta{x}$ = 0.0796 mm (initial mode: HG_1,0_); **b** for low order HG modes generation with $\varDelta{x}$= 0.1909 mm (initial mode: HG_4,0_); **c** for HG_*m*,0_ modes generation between *m* = 10 and *m* = 20 with $\varDelta{x}$ = 0.301 mm (initial mode: HG_9,0_); and **d** for HG_*m*,0_ modes generation between *m* = 21 and *m* = 30 with $\varDelta{x}$ = 0.371 mm and an initial *θ* = 4° (initial mode: HG_20,0_). Note that in each figure, $\varDelta{x}$ is only fixed for the hybrid scheme, and the gain for off-axis pumping and non-collinear pumping is calculated using the optimum $\varDelta{x}$ and *θ* values, respectively associated with each HG mode

A numerical analysis to evaluate the relative performance between the pure translation-based off-axis pumping, pure angle-based non-collinear pumping and hybrid methods was carried out (see “Materials and methods” section). Representative simulation results and a comparison between these three methods are shown in Fig. 7. More detailed simulation results are summarized in Fig. S1 and Fig. S2.

We found that the angle-based non-collinear pumping scheme always provided the lowest gain for each mode order compared to the hybrid and pure translation-based methods. Therefore, we focus on the comparison between the pure translation-based off-axis pumping and the hybrid pumping schemes.

Figure 7a shows the gain calculation starting from $\varDelta{x}$ = 0.0796 mm (an initial mode of HG_1,0_). The gains at different HG_*m*,0_ mode orders (*m* = 2 to *m* = 9) were then calculated with the different pumping geometries. We can see that the pure translation-based off-axis pumping scheme provides larger gain for all the mode orders than the hybrid pumping scheme in this case. We increased the starting *∆x* value to 0.1909 mm (an initial mode of HG_4,0_) and compared the gains for HG_*m*,0_ modes with orders from *m* = 5 to *m* = 12 (Fig. 7b). The hybrid pumping scheme shows an advantage for HG_5,0_ to HG_7,0_, but still provides less gain at higher mode order compared to the translation-based off-axis pumping scheme. The gain calculation and comparison with other *∆x* values are shown in Fig. S1. We can find that for low initial mode orders (*m* < 10), the hybrid pumping scheme either cannot provide higher gain or provide higher gain only within a small range of mode orders (corresponding to a small tuning range of *θ* values) compared to the translation-based off-axis pumping scheme.

Although we can alternately tune the $\varDelta{x}$ and *θ* frequently to maintain the gain advantage of the hybrid pumping scheme, it makes the continuous tunability of mode orders more difficult and complicates achieving mode locking. Furthermore, in the small order range where the hybrid scheme is advantageous, the gain difference is only marginal. Therefore, we choose to generate HG_*m*,0_ modes with *m* = 1 to *m* = 10 directly with the translation-based off-axis pumping scheme in the experiment.

On the other hand, for generating high order HG_*m*,0_ modes (*m* ≥ 10), the advantage of the hybrid pumping scheme becomes significant compared to the translation-based off-axis pumping scheme within a broad range of mode orders (Fig. 7c, d). This higher gain is also beneficial for achieving mode locking. Therefore, we generated HG_*m*,0_ modes with *m* ≥ 10 with the hybrid pumping scheme in the experiment.

### Generation of FOVs with mode conversion

**Fig. 8:**
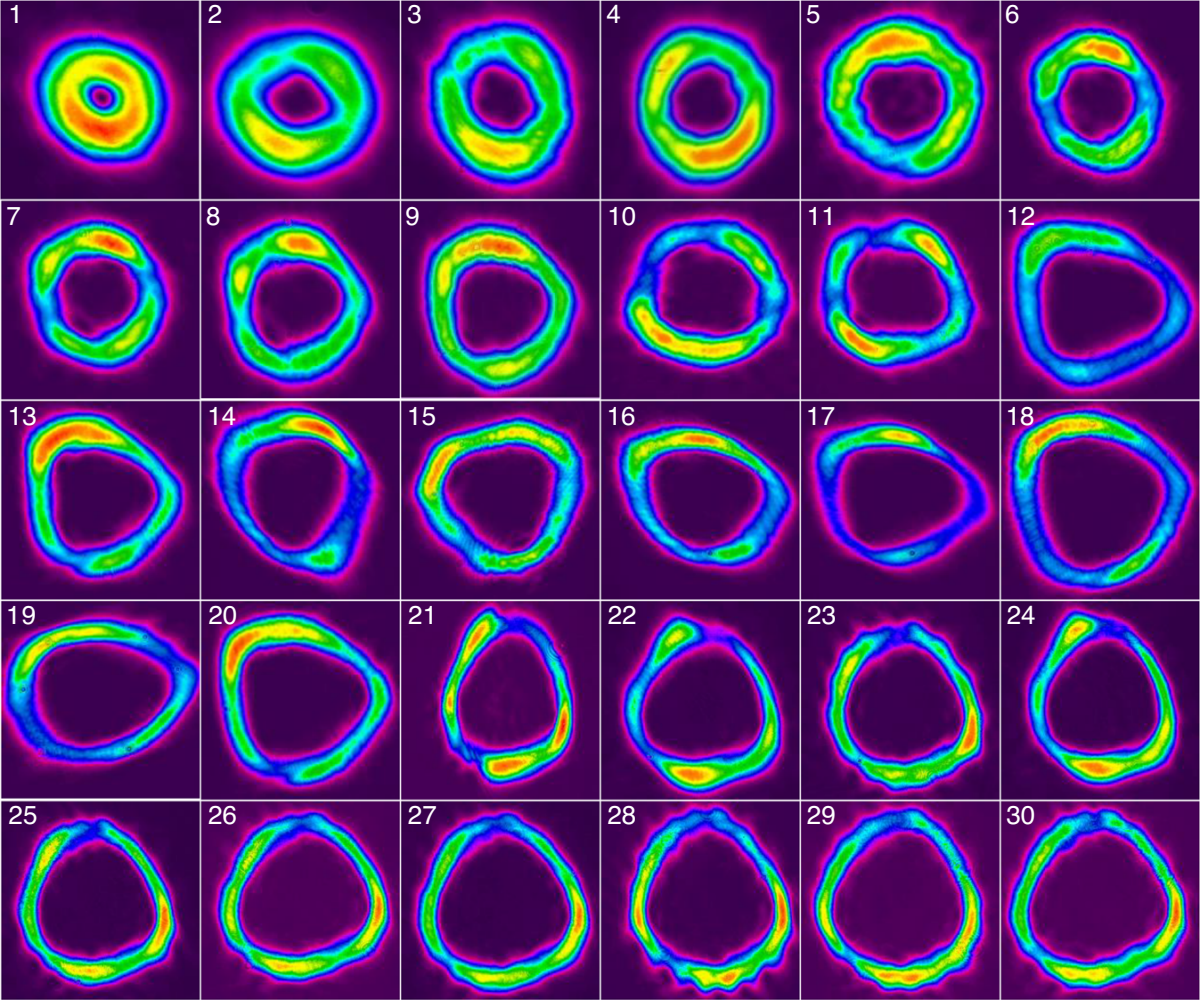
Beam profiles of the femtosecond LG_0,*n*_ vortices. Beam profiles of the femtosecond LG_0,*n*_ vortices obtained by converting the corresponding HG modes from the 1st up to the 30th order

**Fig. 9:**
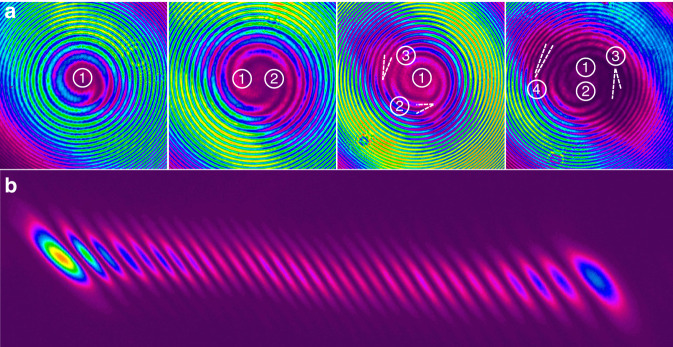
Interference patterns and back converted mode. **a** Interference patterns of the spatial phases measured for LG_0,1_, LG_02_, LG_0,3_ and LG_0,4_, respectively, from left to right, with the furcation indicated in the center. **b** Beam profile of the HG mode obtained by back converting the LG_0,30_ beam, showing a topological charge of 30

Finally, mode conversion from femtosecond HG modes to femtosecond LG modes was achieved in an AMC stage that consisted of a focusing lens followed by a pair of uncoated cylindrical lenses. The beam profiles of the resulting FOVs are shown in Fig. 8. The AMC stage introduced a loss of roughly 10% of the average power but has almost no effect on the pulse duration for our sub-650-fs pulses (Table 1). In order to analyze the phase singularity and the topological charge number of the LG modes, we used a home-built Mach-Zehnder interferometer to characterize the spatial phase properties by inspecting the interference pattern between the vortex beam and a spherical wave created by a focusing lens (Fig. 2a). The phase interference patterns for the LG modes from LG_0,1_ to LG_0,4_ are shown in Fig. 9a, exhibiting a clear furcation around the center with a furcation number from 1 to 4, respectively. This proves that the generated FOVs have spiral phases with topological charges of 1–4 for the LG modes from LG_0,1_ to LG_0,4_, respectively. For higher-order LG mode beams, the furcation was not clearly visible in the phase interference patterns due to the exceedingly large spot size and high fringe density. Instead, the LG mode beam was reflected back through the AMC stage and converted into a HG mode beam again. The topological charge number of the LG mode can then be identified by counting the nodal lines of the HG mode which it has been converted into^35^. Figure 9b shows the profile of the obtained HG mode beam converted back from an LG_0,30_ beam, where 30 nodal lines can be clearly identified.

## Discussion

Our FOV source can be used to develop femtosecond optical tweezers. Optical trapping of particles usually results from an interplay between the gradient and scattering forces caused by electromagnetic fields^36^. These are closely related to the refractive index of the captured particle. When a femtosecond beam is used, its extremely high intensity can nonlinearly alter the local refractive index of the trapped particles, thus providing extra degrees of freedom for their capture and control. At the same time, the orbital angular momentum carried by a vortex beam can rotate the trapped particles^37^. The velocity of the rotation is proportional to the topological charge—the larger the topological charge, the faster the rotation speed. Thus, the development of high topological charge femtosecond vortex light can promote the further development of femtosecond vortex optical tweezers.

Moreover, this source also has potential applications in material processing. The reduced thermal diffusion and lack of heat-affected zone formation, combined with the nonlinear absorption dynamics (i.e., multi-photon absorption) allow high-quality micro-machining even inside of transparent materials^38^. Adding vortex characteristics to the beam enables the laser pattern to be structured more flexibly^39^ and allows generating 3D chiral microstructures^40^. Therefore, a continuously tunable femtosecond vortex beam will find considerable applications in microstructure machining.

In order to generate FOVs with orders higher than 30, more pump power is required to compensate for the increased diffraction loss. Although no sign of damage to the Yb:KGW crystal has been observed yet, the risk of crystal damage would be greatly increased with higher pump power. An optimized cooling setup would be beneficial to pump more strongly, as well as for preventing thermal effects that are detrimental to the mode locking. In addition, the switching between displacement and angle tuning needs to be further investigated in order to optimize the conditions for longitudinal mode locking, which is instrumental for the generation of higher-order femtosecond vortices.

The current pulse duration of FOV beams is limited mainly by the imperfect dispersion compensation provided by a single GTI mirror, whose large GDD of −2000 fs^2^ limited its supported bandwidth. This can be improved in the future by replacing the single GTI mirror with multiple mirrors with lower GDD but broader bandwidth. Besides, the total GDD compensation can also be finely tuned to shorten the pulse duration. Furthermore, gain crystals with a broader emission spectrum such as Yb:CALGO will be also tested for the generation of shorter HG pulses. Sub-100 fs pulse duration can be expected by implementing these measures.

In addition, the beam profiles of the HG mode beams need to be further improved since they are crucial to the beam quality of the FOVs. The concave mirrors used in the current oscillator introduced cavity astigmatism to the beam profiles of the HG modes and consequently to the FOVs. According to ref. ^33^, the astigmatism can be compensated by creating two planes perpendicular to each other, which is realized by increasing the height of the plane mirrors next to the concave mirrors comprising the telescope section inside the oscillator cavity. A better beam quality can be expected by utilizing this method in the future.

In conclusion, we have demonstrated the generation of continuously tunable high-power, high-order femtosecond HG pulses from the 1st to the 30th order using a SESAM mode-locked Yb:KGW laser oscillator. FOVs were then generated by converting the HG pulses in an AMC, demonstrating the highest vortex order obtained to date from any femtosecond vortex laser source based on the mode-locked oscillators. This powerful tunability of the FOVs originating from a mode-locked oscillator is made possible by employing a hybrid scheme consisting of translation-based off-axis pumping and angle-based non-collinear pumping techniques. The pulses at all vortex orders have average powers of several hundred milliwatts and pulse durations of <650 fs. Notably, the average power of the LG_0,11_ mode beam could reach 1.6 W, which is, to our knowledge, the highest among state-of-the-art oscillator-based femtosecond vortex sources. Our scheme is also applicable to other types of gain media, which may lead to new records in the generation of ultrashort high-order optical vortex pulses, providing a potential platform for novel and interesting studies of light-field control with multi-dimensional tunability.

## Materials and methods

### Oscillator cavity design and realization of mode locking

The oscillator was designed based on the ABCD matrix method. We calculated the beam size inside the cavity in the case of the fundamental transverse mode. The beam waist diameter of the fundamental laser mode inside the crystal was ~100 μm, comparable with the pump beam size. To achieve a high power density on the SESAM and initiate the passive mode locking, we placed a concave mirror (M1) with a radius of curvature of -150 mm in front of the SESAM (Fig. 2a). The beam diameter on the SESAM was designed and calculated as 80 μm (fundamental transverse mode). During the experiment, the distances within the telescope section (M2 to M3) and between the M1 and SESAM were precisely tuned. Passive mode locking could be initiated automatically when the pump power was increased to certain values for the HG modes at different orders.

### Numerical model of the hybrid scheme

We have built a numerical model to analyze the pump threshold of the HG_*m*,0_ modes with the hybrid non-collinear scheme. It is an improvement of the model reported in ref. ^30^, which applies to the purely angle-based non-collinear scheme. The normalized pump beam in the gain medium can be described as

1 $${r}_{p}(x,y,z)=\frac{2}{\pi {w}_{x}(z){w}_{y}(z)}\frac{\alpha }{1-{e}^{-\alpha L}}\exp \left[-\frac{2{x}^{2}}{{w}_{x}^{2}(z)}-\frac{2{y}^{2}}{{w}_{y}^{2}(z)}-\alpha z\right]$$

where ${w}_{x}\left(z\right)$ and ${w}_{x}\left(z\right)$ are the spot radii of the pump beam in the *x* and *y* directions, respectively, and $\alpha$ is the absorption coefficient of the gain medium at the pump wavelength. Based on this formula, a new variable ${x}_{i}$ is introduced to replace $x$, with the following relationship between the two variables:

2 $${x}_{i}=x+\varDelta x$$

Then, the pump threshold for HG_*m*,0_ can be expressed as:

3 $${P}_{{\rm{th}}}\left({{\rm{HG}}}_{m,0}\right)=\frac{\gamma {I}_{{\rm{sat}}}}{{n}_{p}L}\frac{1}{\int\!\!\int\!\!\int {s}_{m,0}({x}^{{\prime} },{y}^{{\prime} },{z}^{{\prime} }){r}_{p}({x}_{i},y,z){dxdydz}}$$

Here, $\gamma$ is the total loss coefficient of the laser cavity, *I*_sat_ is the saturation intensity, *L* is the length of the gain medium, and *n*_*p*_ is the pumping efficiency. *s*_*m,0*_ (*x'*, *y'*, *z'*) is the normalized laser intensity distribution in the gain medium, where (*x'*, *y'*, *z'*) is a rotationally transformed coordinate system by an angle *θ* and *r*_*p*_ (*x*_*i*_, *y*, *z*) is the normalized pump intensity distribution as defined above (see Eq. (1)). As a result, the pump threshold for HG_*m*,0_ modes can be obtained from *m* = 9 to *m* = 21 and from *m* = 20 to *m* = 31 as a function of the angle *θ* at a fixed off-axis displacement (*∆x* = 0.301 mm and 0.371 mm, respectively).

### Gain calculation for different pumping schemes

The gain obtained by different modes can be simply described as

4 $$G({{\rm{HG}}}_{m,0})=\int\!\!\int\!\!\int {s}_{m,0}({x}^{{\prime} },{y}^{{\prime} },{z}^{{\prime} }){r}_{p}({x}_{i},y,z){dxdydz}$$

For low-order HG_*m*,0_ modes (*m* < 10), we first determined the *∆x* values (slightly different from the actual values in the experiment as shown in Fig. 4c) that provided the optimum gain for each higher-order mode using the pure translation method (*θ* = 0). For each resulting *∆x*, we then simulated the gain for different higher-order modes when various *θ* values are included, i.e. using the hybrid scheme. Note that each *∆x* value is uniquely associated with an optimum pure-translation-based higher-order mode which is the initial mode. But under the hybrid scheme, when *θ* is varied, the same *∆x* can lead to different higher-order modes with varying performance. The results for progressively larger *∆x* and their combination with different *θ* values are plotted in Fig. S1. For completeness, we have also calculated the maximum achievable gain for each higher-order mode when *∆x* is zero and only *θ* is adjusted.

For HG_*m*,0_ modes with *m* from 10 to 20, the method of gain calculation is the same as in the case of low-order HG_*m*,0_ modes. However, we use the actual *∆x* value (0.301 mm in the experiment) to get the initial mode of HG_9,0_. It shows the same tendency of the gain compared to the result obtained from the optimum *∆x* value. For HG_*m*,0_ modes (*m* = 21 to *m* = 30), the initial mode HG_20,0_ is determined by both *∆x* (0.371 mm) and an initial *θ* = 4° in order to match with the actual experimental parameters. In this case, the gain value of each mode under the hybrid pumping scheme is different from the result calculated with only a single optimum *∆x* value, but the gain tendency is the same within the order range from *m* = 21 to *m* = 30. With such a large *∆x* value, the hybrid pumping scheme shows higher gain than the other two schemes within a broad range of mode orders (corresponding to a broad tuning range of *θ* values). We do not introduce an initial angle for small *∆x* values (corresponding to lower-order initial HG modes) since the tuning range of *θ* values is limited.

### Conversion from the HG mode to the LG mode with an AMC

An AMC system consisting of two cylindrical lenses and a convex lens was used to convert the HG mode to the LG mode. The principle of mode conversion by an AMC has been described in ref. ^28^. The input HG mode can be decomposed into a set of HG modes of the same order:

5 $${u}_{{nm}}^{{\rm{HG}}}\left(\frac{x+y}{\sqrt{2}},\,\frac{x+y}{\sqrt{2}},\,z\right)=\mathop{\sum }\limits_{k=0}^{N}\,b\left(n,\,m,\,k\right){u}_{N-k,k}^{{HG}}(x,\,y,\,z)$$

where *N* = *n* + *m*, and the coefficient

6 $$b(n,m,k)={\left(\frac{(N-k)!k!}{{2}^{N}n!m!}\right)}^{1/2}\times \frac{1}{k!}\frac{{d}^{k}}{d{t}^{k}}\left[{(1-t)}^{n}{(1+t)}^{m}\right]_{t=0}$$

After passing the AMC, a relative phase difference of π/2 is introduced between the successive components. The LG mode can be composed by combining these different components, which can be described as:

7 $$\mathop{\sum }\limits_{k=0}^{N}\,{i}^{k}b(n,\,m,\,k)\,{u}_{N-k}^{{\rm{HG}}}(x,\,y,\,z)={u}_{{pl}}^{{\rm{LG}}}(x,\,y,\,z)$$

where the index *p* is the minimum of *n* and *m* (min (*n*, *m*)) and the index *l* is the absolute value of *n−m* (|*n−m*|). This way, an HG mode can be converted into an LG mode by an AMC.

### Supplementary information

Supplementary Materials for High-order femtosecond vortices up to the 30th order generated from a powerful mode-locked Hermite-Gaussian laser

## Data Availability

All data are available from the corresponding authors upon reasonable request.
