# Supplementary material for: High-order femtosecond vortices up to the 30th order generated from a powerful mode-locked Hermite-Gaussian laser
Source: Light Sci Appl. 2023 Aug 30;12:207. doi: 10.1038/s41377-023-01241-z (PMC10469186; doi:10.1038/s41377-023-01241-z)
Supplement: Supplementary file 1 — Supplementary Materials for High-order femtosecond vortices up to the 30th order generated from a powerful mode-locked Hermite-Gaussian laser [file 41377_2023_1241_MOESM1_ESM.docx]

**Supplementary**

**High-order femtosecond vortices up to the 30th order generated from a powerful mode-locked Hermite-Gaussian laser**

Hongyu Liu^1,†^, Lisong Yan^1,†^, Hongshan Chen^1^, Xin Liu^1^, Heyan Liu^1^, Soo Hoon Chew^2,3^, Alexander Gliserin^2,3^, Qing Wang^4,*^, and Jinwei Zhang^1,*^

1, School of Optical and Electronic Information and Wuhan National Laboratory for Optoelectronics, Huazhong University of Science and Technology, Wuhan 430074, China

2, Department of Optics and Mechatronics Engineering, College of Nanoscience and Nanotechnology, Pusan National University, Busan 46241, South Korea

3, Max Planck Center for Attosecond Science, Max Planck POSTECH/Korea Research Initiative, Pohang 37673, South Korea

4, School of Optics and Photonics, Beijing Institute of Technology, Beijing 100081, China


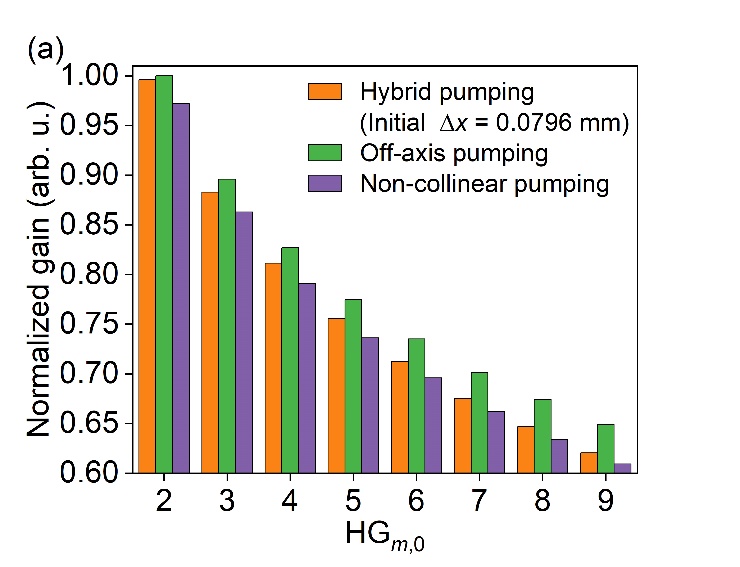

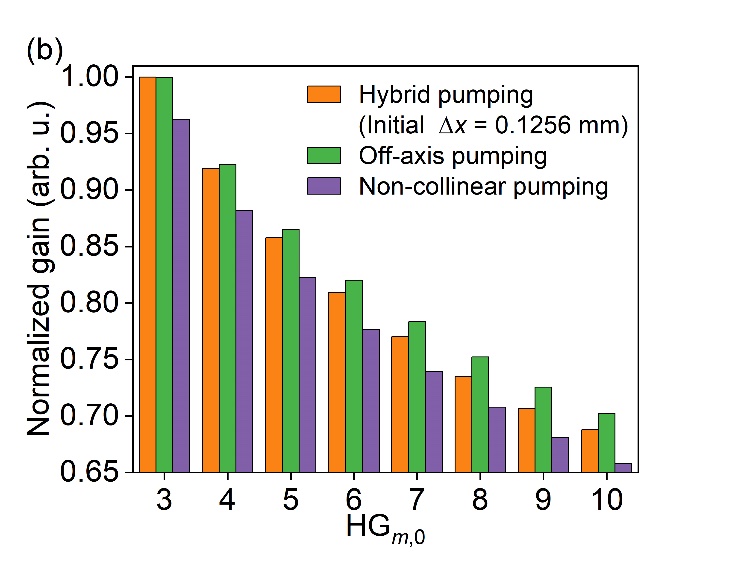


(a) *∆x* = 0.0796mm (initial mode: HG_1,0_) (b) *∆x* = 0.1256 mm (initial mode: HG_2,0_)


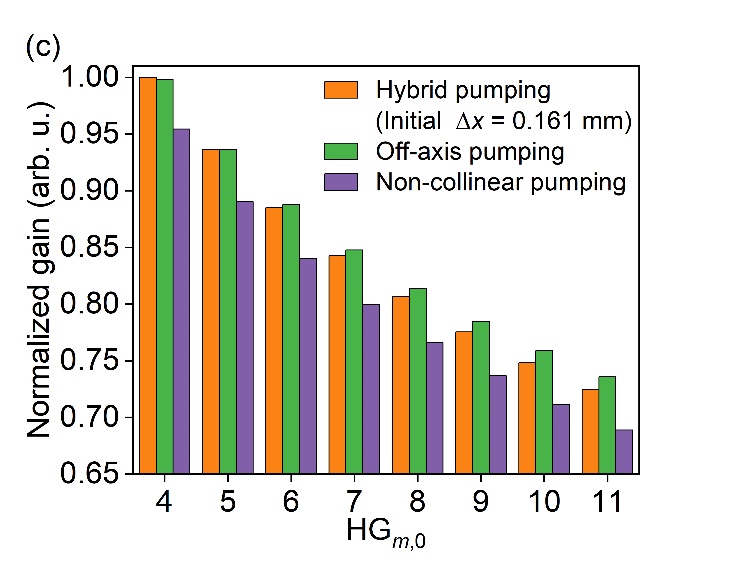

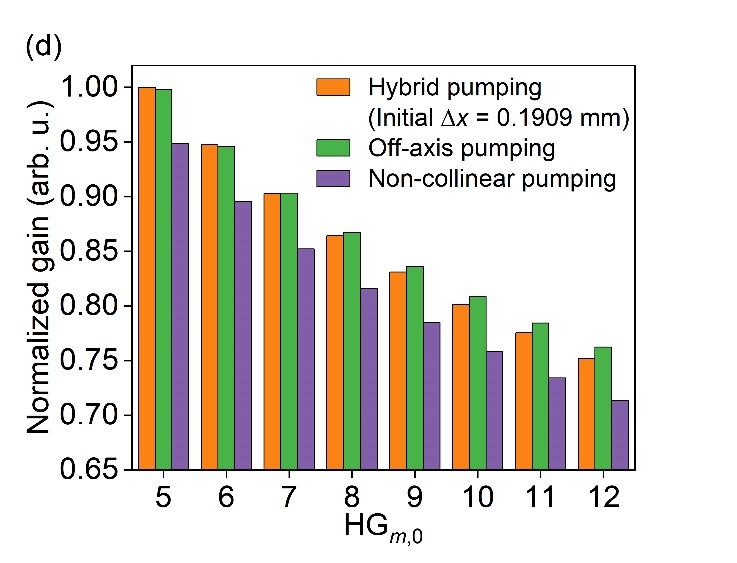


(c) *∆x* = 0.161 mm (initial mode: HG_3,0_) (d) *∆x* = 0.1909 mm (initial mode: HG_4,0_)


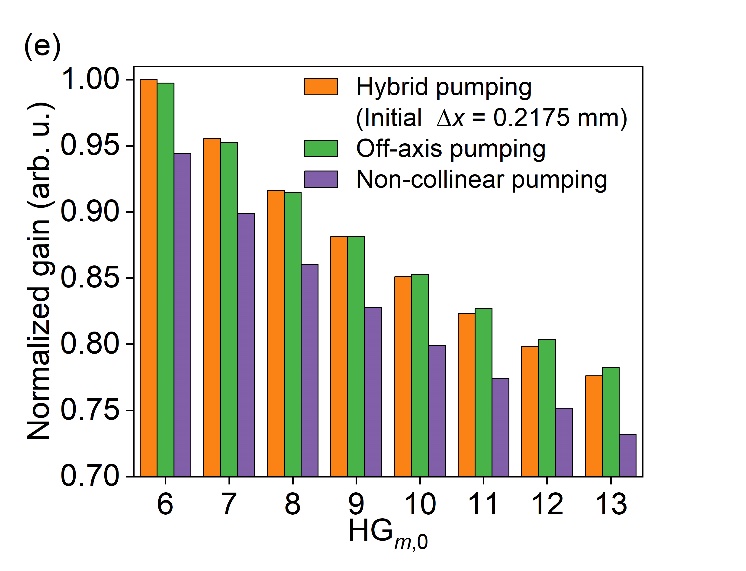

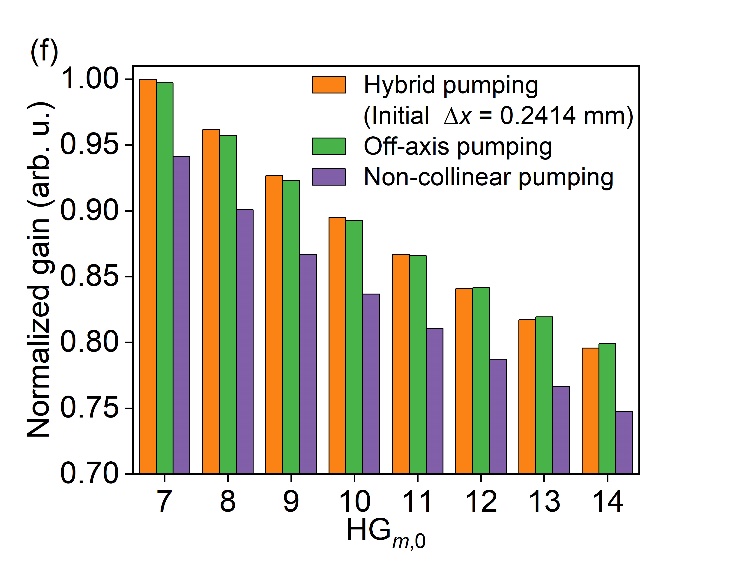


(e) *∆x* = 0.2175 mm (initial mode: HG_5,0_) (f) *∆x* = 0.2414 mm (initial mode: HG_6,0_)


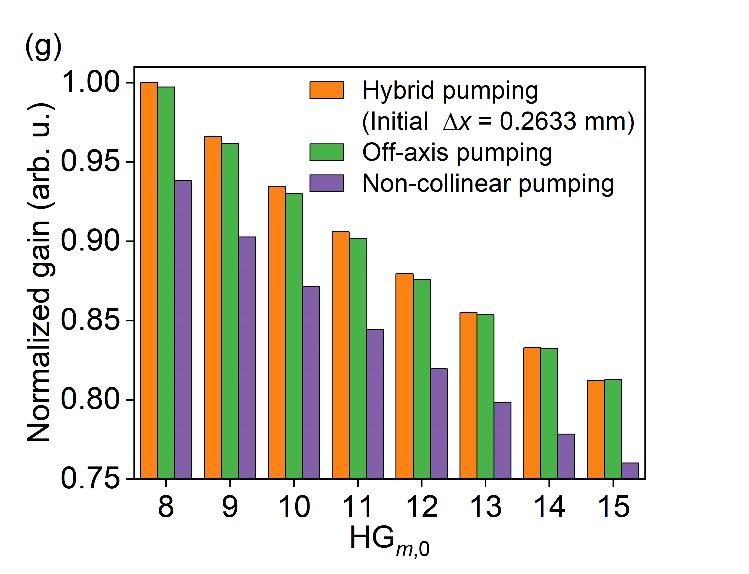

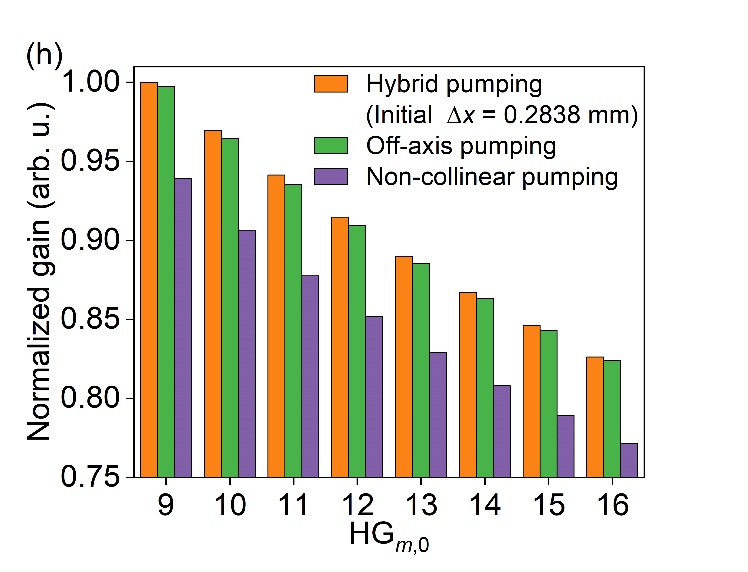


(g) *∆x* = 0.2633 mm (initial mode: HG_7,0_) (h) *∆x* = 0.2838 mm (initial mode: HG_8,0_)

Fig. S1. Gain calculation and comparison for low order HG modes generation with different starting *∆x* values by the three methods (pure translation-based off-axis pumping, pure angle-based non-collinear pumping, and hybrid pumping scheme). (a) *∆x* = 0.0796 mm (initial mode: HG_1,0_); (b) *∆x* = 0.1256 mm (initial mode: HG_2,0_); (c) *∆x* = 0.161 mm (initial mode: HG_3,0_); (d) *∆x* = 0.1909 mm (initial mode: HG_4,0_); (e) *∆x* = 0.2175 mm (initial mode: HG_5,0_); (f) *∆x* = 0.2414 mm (initial mode: HG_6,0_); (g) *∆x* = 0.2633 mm (initial mode: HG_7,0_); (h) *∆x* = 0.2838 mm (initial mode: HG_8,0_). We show the comparison of the successive eight mode orders for each initial mode (*∆x* value). Note that in each figure, *∆x* is only fixed for the hybrid scheme, and the gain for off-axis pumping and non-collinear pumping is calculated using the optimum *∆x* value and *θ* value, respectively associated with each HG mode.


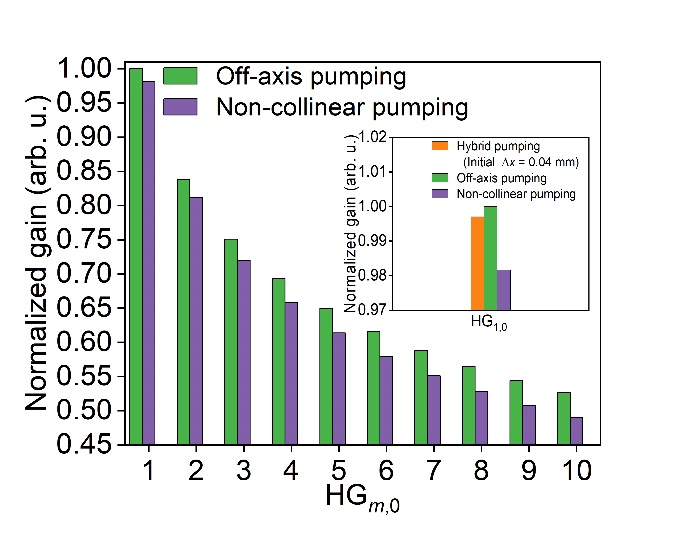


Fig. S2. Gain calculation and comparison for low order HG modes (from *m* = 1 to *m* = 10) generated by pure translation-based off-axis pumping and pure angle-based non-collinear pumping scheme. Note that the HG_1,0_ mode can also be generated by tuning both *∆x* and *θ* together, however, the gain will be lower than that obtained from pure translation-based off-axis pumping scheme (Inset).


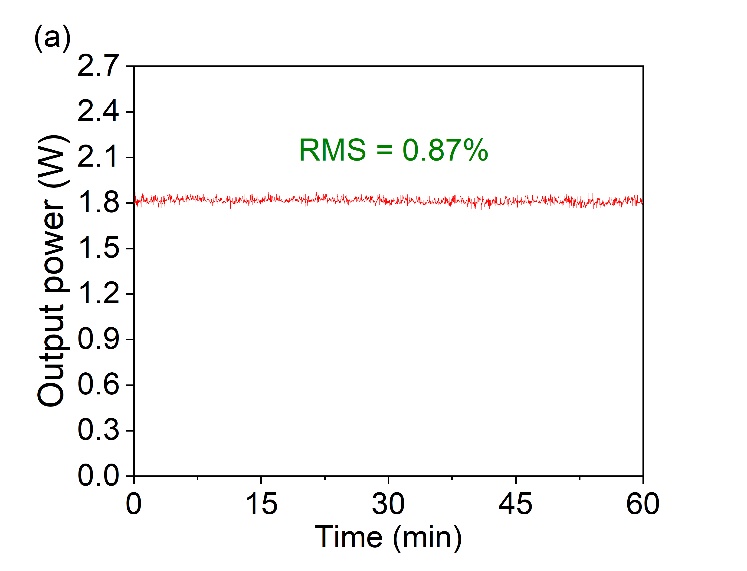

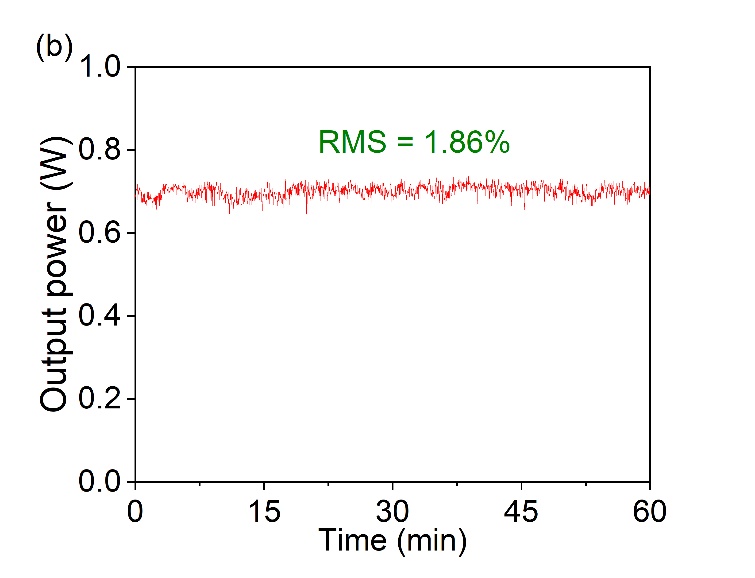


Fig. S3. Power stability measurement. (a) Power fluctuations of HG_11,0_ within one hour, showing a deviation of 0.87% (RMS); (b) Typical power deviation of high-order HG*_m_*_,0_ modes (from *m* = 20 to *m* = 30), which is a bit larger than low-order HG*_m_*_,0_ modes.


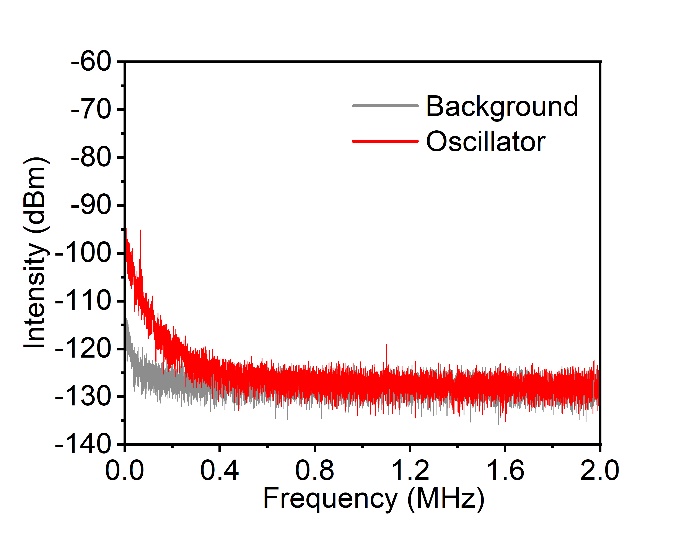


Fig. S4. Qualitative intensity noise measurement by radio frequency (RF) spectrum analyzer from 10 Hz to 2 MHz with a resolution bandwidth (RBW) of 51 Hz (averaged 10 times). The calculated RMS value of relative intensity noise is about 0.43%.
